# Targeted deletion of grape retrotransposon associated with fruit skin color via CRISPR/Cas9 in *Vitis labrascana* ‘Shine Muscat’

**DOI:** 10.1371/journal.pone.0286698

**Published:** 2023-06-08

**Authors:** Ikuko Nakajima, Hiroyuki Kawahigashi, Chikako Nishitani, Akifumi Azuma, Takashi Haji, Seiichi Toki, Masaki Endo

**Affiliations:** 1 Institute of Fruit Tree and Tea Science, National Agriculture and Food Research Organization, Tsukuba, Japan; 2 Institute of Agrobiological Sciences, National Agriculture and Food Research Organization, Tsukuba, Ibaraki, Japan; 3 Faculty of Agriculture, Ryukoku University, Otsu, Shiga, Japan; 4 Graduate School of Nanobioscience, Yokohama City University, Yokohama, Kanagawa, Japan; Texas Tech University, UNITED STATES

## Abstract

Transposition of transposable elements affect expression levels, splicing and epigenetic status, and function of genes located in, or near, the inserted/excised locus. For example, in grape, presence of the *Gret1* retrotransposon in the promoter region of the *VvMYBA1a* allele at the *VvMYBA1* locus suppress the expression of the *VvMYBA1* transcription factor gene for the anthocyanin biosynthesis and this transposon insertion is responsible for the green berry skin color of *Vitis labrascana*, ‘Shine Muscat’, a major grape cultivar in Japan. To prove that transposons in grape genome can be removed by genome editing, we focused on *Gret1* in the *VvMYBA1a* allele as a target of CRISPR/Cas9 mediated transposon removal. PCR amplification and sequencing detected *Gret1* eliminated cells in 19 of 45 transgenic plants. Although we have not yet confirmed any effects on grape berry skin color, we were successful in demonstrating that cleaving the long terminal repeat (LTR) present at both ends of *Gret1* can efficiently eliminate the transposon.

## Introduction

Grape is an important fruit crop with a large market for both table grapes and wine. *Vitis labrascana*, ‘Shine Muscat’ is a diploid grape cultivar bred at the Grape Research Center of the National Institute of Fruit Tree Sciences (NIFTS), Japan [1, 2]. The fruit is green and sweet at maturity, with high sugar and low acid contents. It has a muscat flavor, with crisp and juicy flesh. It has a degree of disease resistance to downy mildew and ripe rot, inherited from the parent American parents, and resistance to cold weather [1, 3]. Even in hot summers, its color does not deteriorate. It can be eaten with its skin intact, and is seedless following gibberellin treatment, with taste, texture, and aroma comparable to those of European grapes. It is very popular in Japan, where its cultivation area is increasing [4], and in East Asia owing to its excellent storability.

RNA-guided genome editing using the CRISPR (clustered regularly interspaced short palindromic repeats)/Cas9 (CRISPR-associated protein 9) system has been applied successfully in several tree fruit species; for example, apple [5], kiwifruit [6], citrus [7], and banana [8]. In grape, there are nine reports of genome editing using CRISPR/Cas9 introduced via *Agrobacterium*-mediated transformation [9–17].

CRISPR/Cas9 induces DNA double-strand breaks at specific sites in the genome. These breaks have a high propensity to induce site-directed mutations through error-prone genome repair due to non-homologous end-joining. The induction of point mutations in specific genes via CRISPR/Cas9 has advantages over crossbreeding and Targeting Induced Local Lesions in Genomes (TILLING). In addition, CRISPR/Cas9 can induce frame-shift mutations that inactivate the target genes. Because fruit trees are genetically heterogeneous, CRISPR/Cas9 is a promising tool for adding precise characteristics to existing varieties.

Fruit color is due to the synthesis of anthocyanins in the skin. There are two functionally important grape berry pigmentation loci: *VvMYBA1* (with alleles *VvMYBA1a*, *VvMYBA1b*, and *VvMYBA1c*) and *VvMYBA2* (with alleles *VvMYBA2r* and *VvMYBA2w*), on chromosome 2. Expression analysis of MYBA genes showed that *VvMYBA1* and *VvMYBA2* are highly expressed in berry pericarp and berry skin, but barely expressed in other tissues [18]. *VvMYBA1a* and *VvMYBA2w* are loss-of-function alleles [19]. These two loci are inherited together and are considered part of a single large allele (haplotype) [20]. The combination of their alleles determines the berry color: no functional alleles, green; one functional allele, red; two functional alleles, purple or black.

**Fig 1:**
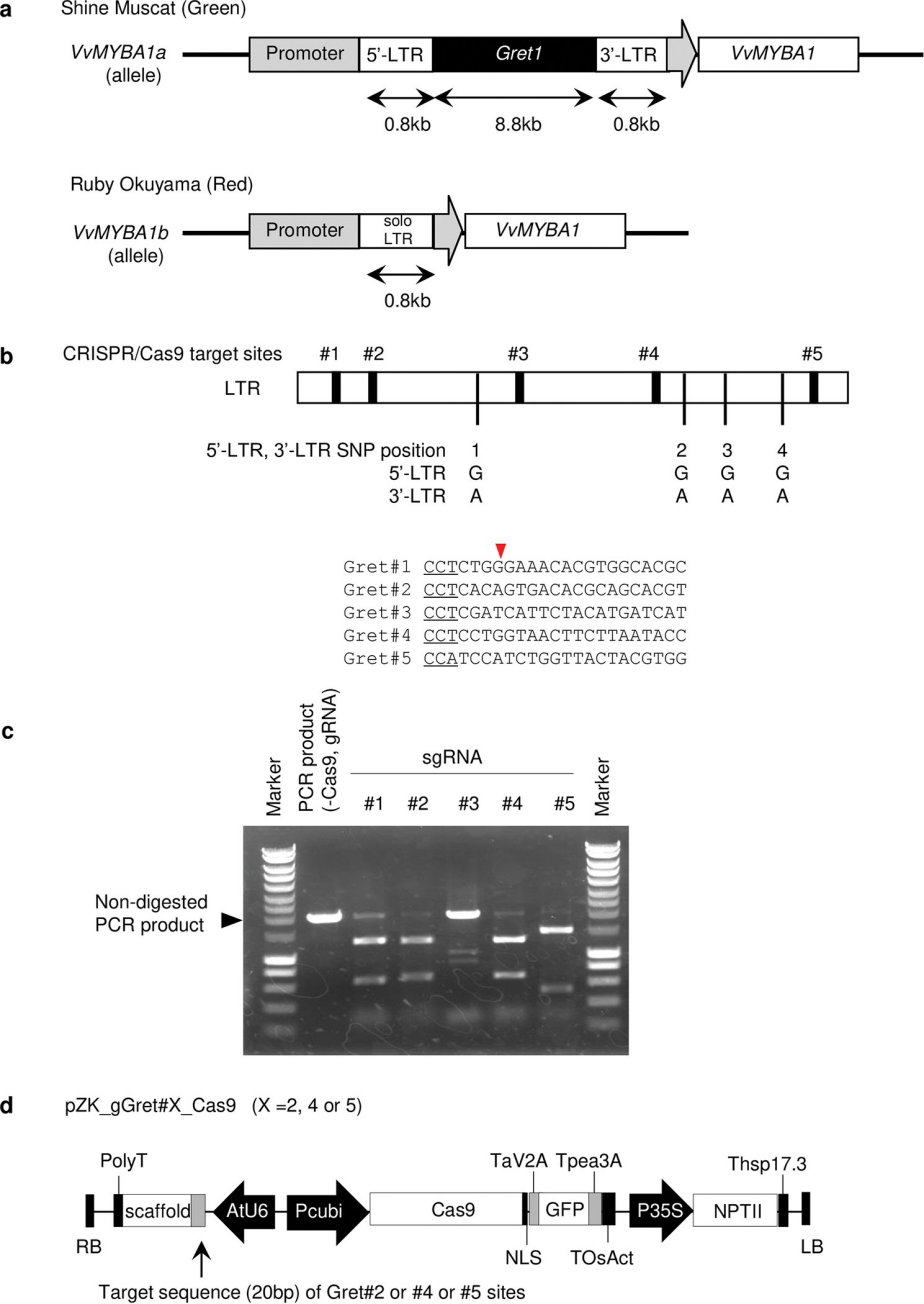
Schematic figure of *VvMYBA1* locus and the peripheral sequences associated with grape skin color. (a) Schematic diagram of *VvMYBA1* locus in ‘Shine Muscat’ and ‘Ruby Okuyama’. In ‘Shine Muscat’, a ~10 kb *Gret1* transposon is inserted in the promoter region. This insertion suppresses *VvMYBA1* expression, resulting in green skin. In ‘Ruby Okuyama’, a solo long terminal repeat (LTR) region remains in the promoter region, but *VvMYBA1* is expressed, resulting in red skin. (b) Schematic diagram of CRISPR/Cas9 target position and the SNPs between 5′-LTR and 3′-LTR in ‘Shine Muscat’. Black boxes, sgRNA target sites (detailed sequences below). CRISPR/Cas9 target sequences exist on the non-coding strand. Underline, sequences complementary to PAM sequences (CCN) on the coding strand; Red arrowhead, expected CRISPR/Cas9 cleavage position; Vertical bars, SNP positions (1–4) between 5′-LTR and 3′-LTR, with nucleotides shown. (c) *in vitro* cleavage assay using PCR product, Cas9 protein, and sgRNAs. Black arrowhead, non-digested PCR products. (d) Expression constructs for Cas9, sgRNA, and selection marker, NPTII. RB, right border; PolyT, polyT terminator; AtU6, Arabidopsis U6-26 promoter; scaffold, trans-activating CRISPR RNA from *Streptococcus pyogenes*; Pcubi, parsley polyubiquitin promoter; NLS, nuclear localization signal; TaV2A, *Thosea asigna* virus 2A peptide; GFP, *Aequrea* green fluorescent protein; Tpea3A, pea rbcS-3A terminator; TOsAct, *Oryza sativa* actin terminator; P35S, cauliflower mosaic virus 35S promoter; NPTII, neomycin phosphotransferase; Thsp17.3, *Oryza sativa* heat shock protein 17.3 terminator; LB, left border.

Kobayashi et al. [21] suggested that the expression of *VvMYBA1* is blocked in the *VvMYBA1a* allele, the promoter region of which contains *Gret1*, a Ty3-gypsy-type retrotransposon. *VvMYBA1b*, which is expressed, has a single long terminal repeat (a solo LTR), which may have occurred as a result of intra-recombination between 5′-LTR and 3′-LTR regions of *Gret1*, in the 5′-flanking region near the *VvMYBA1* coding region [21, 22]. *VvMYBA1c* completely lacks *Gret1* and is most likely the original sequence before the insertion of *Gret1* [23]. *VvMYBA1a* allele has spread among many green grape cultivars around the world from a single origin [24]. Loss of *Gret1* by spontaneous transposition produces a revertant. For example, the red grape ‘Ruby Okuyama’ is a bud mutation of the green grape ‘Italia’, and the red ‘Flame Muscat’ is a bud mutation of the green ‘Muscat of Alexandria’. In ‘Ruby Okuyama’, only a single LTR region is present in the *VvMYBA1* promoter region (Fig 1A), and *VvMYBA1* is expressed, producing anthocyanin [21]. *Gret1* is 10422 bp long: 5′-LTR of 824 bp, internal region of 8774 bp, and 3′-LTR of 824 bp. The sequences of the two LTRs differ at only four nucleotides [21]. Because ‘Shine Muscat’ has two *VvMYBA1a* alleles and two *VvMYBA2w*, its berry skin is green [19]. To turn the skin red, we focused on removing *Gret1* in *VvMYBA1a* by genome editing.

Because transposons can have undesirable effects, techniques to remove them are needed. Since the CRISPR/Cas9 system can cleave target sites, cleaving multiple locations on the same chromosome can remove sequences between cleavages [25]. In our previous study, we eliminated the rice retrotransposon Tos17 by cleaving 5′- and 3′-LTR of Tos17 [26]. In genome editing, selection markers can be used to identify cells, in which CRISPR/Cas9 vector is stably integrated into the genome. However, regenerated plants often exist in a chimeric state consisting of both non-mutated and mutated cells because genome editing occurs independently within transgenic cells. In this study, we aimed to test the efficacy of CRISPR/Cas9-mediated retrotransposon removal in grapevine by attempting to eliminate Gret1 from the *VvMYBA1a* promoter region in ’Shine Muscat’ by CRISPR/Cas9-induced DNA cleavage.

## Materials and methods

### *in vitro* cleavage assay

All steps were performed according to the manufacturer’s instructions provided in the Guide-it Complete sgRNA Screening System (Takara Clontech, Japan) with following modification; 100 ng of PCR products, 5 ng of synthesized single guide RNA (sgRNA), and 100 ng of Cas9 nuclease were mixed in 10 μL of 1× reaction buffer with 1× BSA. Primers used for amplifying cleavage templates and expected band sizes are as follows: VvMYBA1Pro-F1 and Gret1-R1 (S1 Table), Non-digested, 1977 bp; digested at Gret#1, 676 bp and 1301 bp; digested at Gret#2, 700 bp and 1277 bp; digested at Gret#3, 1062 bp and 915 bp; digested at Gret#4, 1270 bp, 707 bp; Gret#5, 1437 bp, 540 bp.

### Vector construction

Oligonucleotide pairs for the target sequences were annealed, and the resulting fragments cloned into the BbsI site of the sgRNA cloning vector pUC19_AtU6oligo, in which the attL1::AtU6-26::gRNA::PolyT::attL2 fragment from pEn-Chimera [27] accompanying two I-SceI sites at both ends was inserted in SmaI sites of the pUC19 (Nippongene, Japan). To complete the all-in-one binary vector harboring sgRNA, Cas9 and an NPTII expression construct, we used pZK_OsU3gYSA_Cas9, which contains a Cas9 expression cassette prepared from pDe-Cas9 [27], an NPTII expression cassette, and sgRNA expression construct OsU3::gYSA. The sgRNA expression cassette, AtU6-26::gGret#2::PolyT, AtU6-26::gGret#4::PolyT, AtU6-26::gGret#5::PolyT, prepared in pUC19_AtU6oligo, was excised at the I-Sce I sites and replaced by OsU3::gYSA in pZK_OsU3gYSA_Cas9, completing pZK_gGret#2_Cas9 to pZK_gGret#5_Cas9.

### Plant material and transformation of grape with Cas9 and sgRNA expression plasmids

*Vitis labrascana*, ‘Shine Muscat’ is derived from a cross between Akitsu-21 and ‘Hakunan’. One parent line, Akitsu-21 (*V*. *labruscana* × *V*. *vinifera*) was obtained by crossing ‘Steuben’ (*V*. *labruscana*) with ‘Muscat of Alexandria’ (*V*. *vinifera*), and the other parent line, ‘Hakunan’ was derived from a cross between ‘Katta Kurgan’ (*V*. *vinifera*) and ‘Kaiji’ (*V*. *vinifera*) [1, 2]. We induced embryogenic callus formation from flower bud filaments from a dormant cane and conducted highly efficient *Agrobacterium*-mediated genetic transformation of ‘Shine Muscat’ as described [28]. *Agrobacterium* strain LBA4404 harboring the binary plasmid pZK_gGret [#2, #4, or #5]_Cas9 was used to infect embryogenic calluses (ECs). Infected ECs were divided into several clusters of aggregated embryo cells (AECs). AECs were selected on (1) half-strength Murashige and Skoog (MS) medium containing 5% maltose and 0.85% agar supplemented with 1 μM 2,4-D, 15 mg L^−1^ kanamycin and 200 mg L^−1^ cefotaxime for 2 weeks to 1 month, then transferred to (2) 1/2 MS containing 5% maltose and 2% agar supplemented with 1 μM 2,4-D, 25 mg L^−1^ kanamycin and 200 mg L^−1^ cefotaxime for 6 months. To induce transformed plants, AECs were cultured as before but without 2,4-D.

### Detection of solo LTR and CRISPR/Cas9-induced mutations at Gret#4 target site

Genomic DNA was extracted from calluses and regenerated plants with a DNeasy Plant Mini Kit (Qiagen, Germany) according to the supplier’s instructions. To detect genome editing in *Gret1* within the *VvMYBA1a* promoter region, we conducted PCR using primers F1 and R1 to amplify the solo LTR, F1, and 5′-LTR-R to amplify the 5′-LTR, and 3′-LTR-F and R1 to amplify 3′-LTR (S1 Table). To confirm the PCR, primers PDS-MPF1 and PDS-MPR1, which amplify the endogenous *PDS* gene, were added to the PCR mixture. Solo LTR PCR products were extracted from the gel and cloned into a Mighty cloning Reagent set (Blunt end) (Takara, Japan) according to the supplier’s instructions, and cloned PCR products were transformed into *Escherichia coli* DH5α (Takara Clontech, Japan). Plasmids were isolated with a TempliPhi Rolling Circle Amplification Kit (GE Healthcare, UK), and sequenced with a BigDye X-Terminator 3.1 cycle sequencing kit (Applied Biosystems, USA) on an ABI3130x sequencer (Applied Biosystems, USA). We used primers F1 and R1 for sequencing solo LTR, 5′-LTR-R and 3′-LTR-F for the 5′-LTR, and 5′-LTR-F3IN and 3FIN-R for the 3′-LTR (S1 Table). To detect mutations in the *Gret1-*like sequence, we used primers Off3-F, Off3-R, 5′-LTR-F, and 5′-LTR-F3IN for PCR and direct sequencing (S1 Table).

## Results

### *in vitro* cleavage assay and *in vivo* solo LTR detection in transgenic calluses

**Fig 2:**
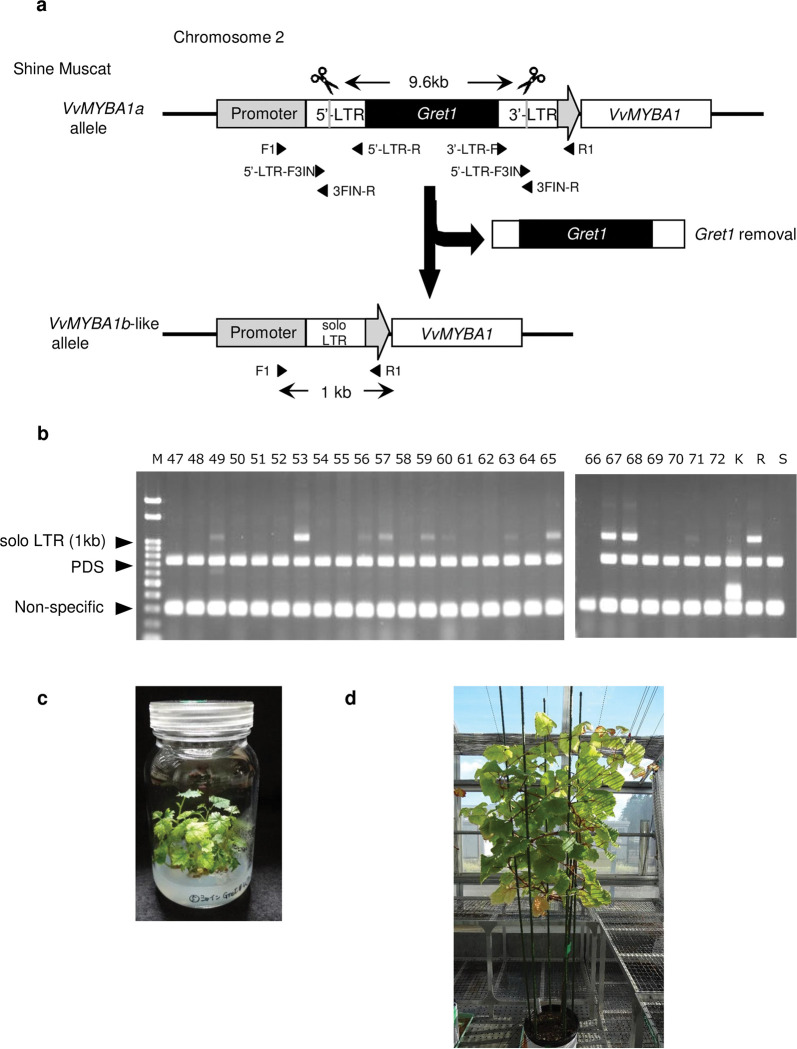
Detection of solo LTR. (a) Schematic diagram of CRISPR/Cas9-mediated Gret1 elimination. 5′-LTR and 3′-LTR regions were cleaved by CRISPR/Cas9 and *Gret1* transposon was removed. Ligation of the two DNA ends results in a single LTR (solo LTR). Black arrowhead: positions and orientations of PCR primers. PCR primers F1 and R1 can amplify the solo LTR DNA fragment (~ 1 kb) when *Gret1* is eliminated. The distance between F1 and R1 in intact ‘Shine Muscat’ is > 10 kb. Under our PCR condition, this long PCR fragment is not amplified. (b) Detection of solo LTR in regenerated plants by PCR using primers F1 and R1. Amplification of 1 kb bands means the existence of solo LTR. Primer pairs amplifying *Vitis vinifera* phytoene desaturase (*VvPDS*) were added to amplify internal control. M, DNA size marker; K, ‘Kyoho’; R, ‘Ruby Okuyama’; S, ‘Shine Muscat’. (c) Regenerated *in vitro* culture of line #67 plantlets. (d) Grafted regenerated #67 plants after nearly 3 years in the greenhouse.

We selected five SpCas9 target sites common in the 5′-LTR and 3′-LTR regions (Fig 1B) and excluded unsuitable sgRNA sequences by *in vitro* cleavage assay. Gret#3 had the poorest result and Gret#1 had slightly more undigested PCR products than Gret#2, #4, and #5 (Fig 1C), so we used Gret#2, #4, and #5 sgRNA for further experiments. CRISPR/Cas9 vectors were prepared (Fig 1D) and transformed into ‘Shine Muscat’. Infected ECs were divided into AECs of ~2 mm: 203 for Gret#2, 202 for Gret#4, and 229 for Gret#5. Selection of these AEC on a kanamycin-containing medium gave 45 transgenic calluses for Gret#2, 44 for Gret#4, and 46 for Gret#5 Following the elimination of *Gret1*, primers F1 and R1 gave solo LTR bands corresponding to 1 kb PCR products (Fig 2A). DNA analysis did not detect any solo LTR band in Gret#2 transgenic calluses, but it was detected in 33 out of 41 Gret#4 transgenic calluses, and in 13 out of 45 Gret#5 calluses (Table 1).

**Table 1:** Detection of solo LTR band of induced transgenic ‘Shine Muscat’ calluses.

Target	No. of AECs	Kanamycin-resistant calluses	PCR-analyzed calluses	Calluses with solo LTR band
Gret#2	203	45	44	0
Gret#4	202	44	41	33
Gret#5	229	46	45	13

### Confirmation of CRISPR/Cas9-mediated *Gret1* elimination in regenerated shoots

As Gret#4 and #5 gave the best results in the *in vitro* cleavage assay and in the detection of solo LTRs in ECs, we used Gret#4 or Gret#5 sgRNA to regenerate plants directly from ECs. Infected ECs were divided into 228 Gret#4 AECs and 239 Gret#5 AECs. We obtained 45 regenerated plants from 7 Gret#4 AECs and 22 plants from 5 Gret#5 AECs. Solo LTR bands were detected in 19 of the 45 Gret#4 plants but were not detected in any of the Gret#5 plants. Detection of the solo LTR is summarized in Table 2, and examples of the multiplex PCR are shown in Fig 2B.

**Table 2:** Detection of solo LTR bands in regenerated plants.

Target	No. of infected AECs	No. of AECs which produced regenerated plants	Regenerated plants	Regenerated plants with solo LTR band
Gret#4	228	7	45	19
Gret#5	239	5	22	0

The solo LTR bands in Gret#4 regenerated plants were subcloned and sequenced to confirm the removal of *Gret1* (Table 3). In 7 out of 11 regenerated plants, 2 or more different solo LTR sequences were identified, indicating sporadic *Gret1* elimination in these plants.

**Table 3:** SNPs and Gret#4 sequences in solo LTR fragments in the regenerated ‘Shine Muscat’ grape plants.

Regenerated plant number	Solo LTR band*	SNPs between 5′-LTR and 3′-LTR^†^					Sequence of Gret#4 in solo LTR PCR fragments	Number of clones	Mutation patterns at Gret#4 target site
			SNP1	SNP2	SNP3	SNP4			
Original sequences		5′-LTR	G	G	G	G	CCTCCTGGTAACTTCTTAATACC		
		3′-LTR	A	A	A	A			
#18	+		G	A	A	A	CCTCCTG----CCTTCTTAATACC	11/11	4 bp deletion
#19	+		G	A	A	A	CCTCCTG-TAACTTCTTAATACC	11/11	1 bp deletion
#27	+		G	A	A	A	CCTCCTGGTAACTTCTTAATACC	5/7	intact Gret#4
			G	A	A	A	CCTCCTG-TAACTTCTTAATACC	1/7	1 bp deletion
			G	A	A	A	CCTCCTGGCAACTTCTTAATACC	1/7	1 bp substitution
#28	+		G	-	A	A	CCTCCT-----------------	10/10	47 bp deletion
#31	+		G	A	A	A	CCTCCT-------TCTTAATACC	11/11	7 bp deletion
#33	+		G	A	A	A	CCTCCT-------TCTTAATACC	11/15	7 bp deletion
			G	A	A	A	CCTCCTGGGTAACTTCTTAATACC	3/15	1 bp insertion
			G	A	A	A	CCTTTT-------TCTTAATACC	1/15	3 bp insertion and 7 bp deletion
#56	+		G	A	A	A	CCTCCTGGTAACTTCTTAATACC	6/11	intact Gret#4
			G	A	A	A	CCTCCTG----CTTCTTAATACC	1/11	4 bp deletion
			G	A	A	A	C-----------TTCTTAATACC	1/11	11 bp deletion
			G	A	A	A	CCT------------------CC	1/11	18 bp deletion
			G	A	A	A	------GTAACTTCTTAATACC	2/11	18 bp deletion
#57	+		G	A	A	A	CCTCCTGGTAACTTCTTAATACC	3/4	intact Gret#4
			G	A	A	A	CCTCCTG-------CTTAATACC	1/4	7 bp deletion
#59	+		G	A	A	A	CCTCCTGGTAACTTCTTAATACC	5/6	intact Gret#4
			G	-	A	A	C----------------------	1/6	49 bp deletion
#65	+		G	A	A	A	CCTCCTGGTAACTTCTTAATACC	1/15	intact Gret#4
			G	A	A	A	CCTCCTG-TAACTTCTTAATACC	1/15	1 bp deletion
			G	A	A	A	CCTCCT--TAACTTCTTAATACC	11/15	2 bp deletion
			G	A	A	A	CCTC-----------TTAATACC	1/15	11 bp deletion
			G	-	-	-	CCTCCT-----------------	1/15	95 bp deletion
#67	+		G	A	A	A	CCTCCTGGGTAACTTCTTAATACC	15/16	1 bp insertion
			G	A	A	A	CTTCCTGGGTAACTTCTTAATACC	1/16	1 bp insertion and 1bp substitution

* “+” Solo LTR fragment was detected.

†“–” Absent owing to sequence deletion.

When the cleaved DNA ends were simply ligated after *Gret1* elimination, SNPs1 to 4 between 5′-LTR and 3′-LTR should be G, A, A, A respectively, because Gret#4 targets sites between SNPs 1 and 2. In most solo LTR PCR products, these SNPs were aligned as expected. If two DNA ends are ligated without insertion, deletion, or substitution, the Gret#4 target sequence is generated again, and the newly created Gret#4 target site can be cleaved by CRISPR/Cas9 again. In fact, both, intact and mutated Gret#4 target sequences were detected in solo LTR in regenerated plants (Table 3).

### Detection of *Gret1* elimination in grafted plants

We grafted *in vitro*-cultured shoots of regenerated plants onto rootstocks and succeeded in grafting 1 shoot from line #5, 1 from line #40, and 3 from line #67.

Solo LTRs were found in all analyzed leaves of #67 grafted plants 1, 2, and 3 (Table 4) but not in the leaves of lines #5 and #40. Unlike before grafting (Table 3, #67), the solo LTRs in grafted plants showed various SNPs patterns with an unmutated Gret#4 target sequence or G- to-T substitution in the Gret#4 target sequence (Table 4).

**Table 4:** SNPs and Gret#4 sequences of solo LTR fragment in different leaves of #67 grafted plants.

#67 grafted plant	Leaf No.	SNPs between 5′-LTR and 3′-LTR				Sequence of Gret#4	Number of clones
		SNP1	SNP2	SNP3	SNP4		
	Expectation	G	A	A	A	CCTCCTGGTAACTTCTTAATACC	
#67–1	1	G	G	A	A	CCTCCTTGTAACTTCTTAATACC	9/10
		G	A	A	A	CCTCCTGGTAACTTCTTAATACC	1/10
	2	G	G	A	A	CCTCCTTGTAACTTCTTAATACC	12/12
	3	G	G	A	A	CCTCCTTGTAACTTCTTAATACC	12/12
	4	G	G	A	A	CCTCCTTGTAACTTCTTAATACC	11/11
	5	G	G	A	A	CCTCCTTGTAACTTCTTAATACC	13/14
		A	A	A	A	CCTCCTGGTAACTTCTTAATACC	1/14
	6	G	G	A	A	CCTCCTTGTAACTTCTTAATACC	7/8
		G	A	A	A	CCTCCTGGTAACTTCTTAATACC	1/8
	7	G	G	A	A	CCTCCTTGTAACTTCTTAATACC	10/10
#67–2	1	G	G	A	A	CCTCCTTGTAACTTCTTAATACC	11/13
		A	G	A	A	CCTCCTTGTAACTTCTTAATACC	1/13
		A	A	A	A	CCTCCTGGTAACTTCTTAATACC	1/13
	2	G	G	A	A	CCTCCTTGTAACTTCTTAATACC	11/13
		A	G	A	A	CCTCCTTGTAACTTCTTAATACC	1/13
		A	A	A	A	CCTCCTGGTAACTTCTTAATACC	1/13
	3	G	G	A	A	CCTCCTTGTAACTTCTTAATACC	16/16
	4	G	G	A	A	CCTCCTTGTAACTTCTTAATACC	13/13
	5	G	G	A	A	CCTCCTTGTAACTTCTTAATACC	9/10
		G	A	A	A	CCTCCTGGTAACTTCTTAATACC	1/10
	6	G	G	A	A	CCTCCTTGTAACTTCTTAATACC	10/11
		A	G	A	A	CCTCCTTGTAACTTCTTAATACC	1/11
#67–3	1	G	G	A	A	CCTCCTTGTAACTTCTTAATACC	11/11
	2	G	G	A	A	CCTCCTTGTAACTTCTTAATACC	7/9
		A	G	A	A	CCTCCTTGTAACTTCTTAATACC	1/9
		A	G	A	A	CCTCCTGGTAACTTCTTAATACC	1/9
	3	G	G	A	A	CCTCCTTGTAACTTCTTAATACC	6/7
		A	A	A	A	CCTCCTGGTAACTTCTTAATACC	1/7
	4	G	G	A	A	CCTCCTTGTAACTTCTTAATACC	9/9
	5	G	G	A	A	CCTCCTTGTAACTTCTTAATACC	11/11
	6	G	G	A	A	CCTCCTTGTAACTTCTTAATACC	6/6

### Detection of mutations in *Gret1* remaining in the *VvMYBA1a* promoter region

**Fig 3:**
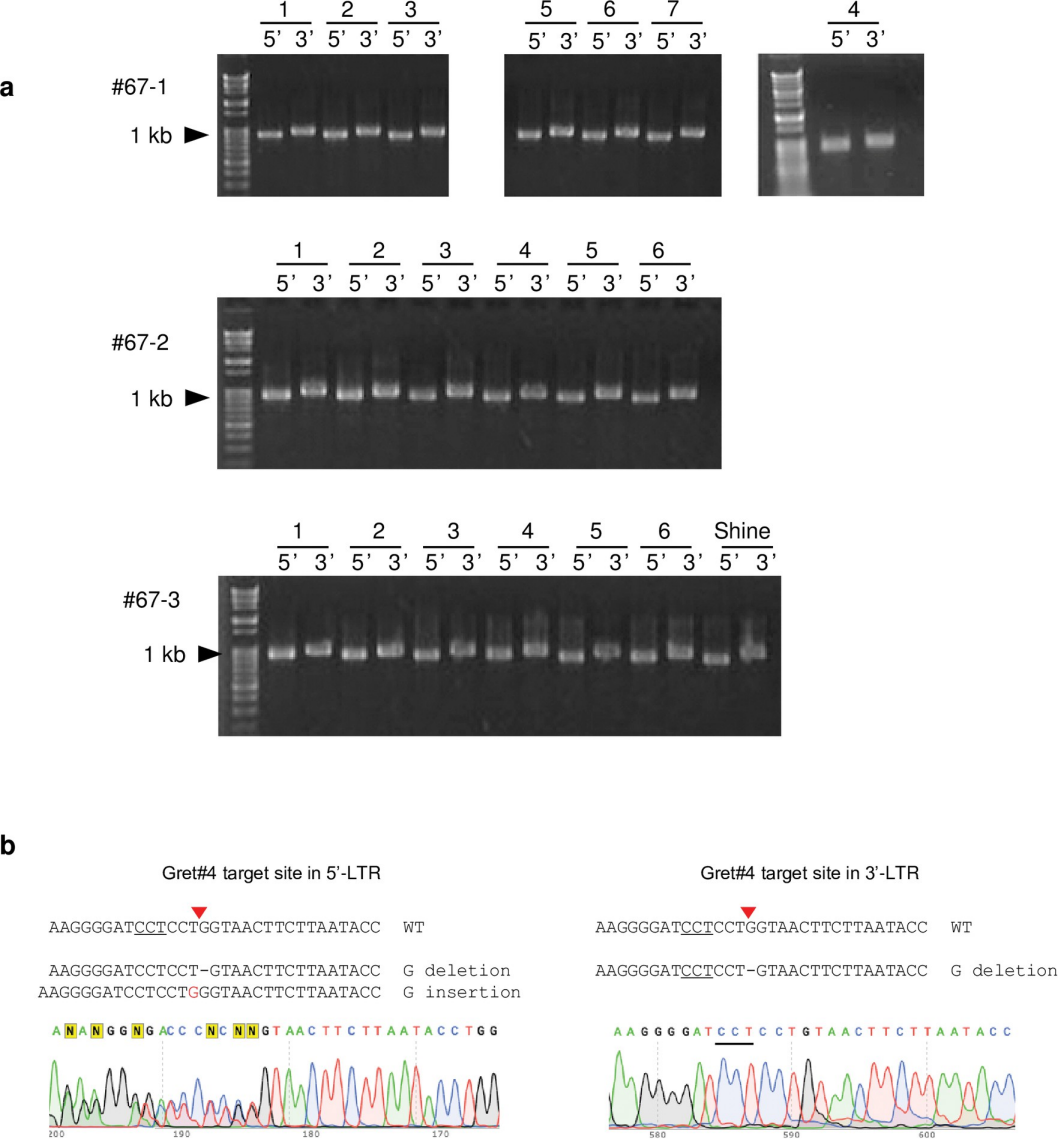
Detection of non-eliminated *Gret1*. (a) PCR amplification of 5′-LTR and 3′-LTR in the leaves of grafted plants #67–1 to 3 using primer sets F1 and 5′LTR-R (5′-LTR), 3′LTR-F and R1 (3′-LTR). (b) Examples of raw sequences of 5′-LTR and 3′-LTR PCR products. All PCR products showed the same sequence patterns except leaf 6 of grafted plant #67–3. In 5′-LTR, double peaks appeared from Gret#4 cleavage site at the ratios close to 1:1. In 3′-LTR, no-double peaks appeared but G deletion was detected.

The detection of diverse SNP variations in solo LTRs in grafted plants and the difference in the Gret#4 target sequences in these solo LTRs from that in the original #67 plant mean that *Gret1* elimination occurred continued to occur sporadically during *in vitro* culture or after grafting, and was not saturated in grafted plants. So we used 5′-LTR- and 3′-LTR-specific PCR to detect the existence of remaining *Gret1*. PCR products of both 5′-LTR and 3′-LTR were detected in all analyzed leaves (Fig 3A). These results mean that the grafted seedlings are in a chimeric state of *Gret1*-removed cells and *Gret1*-residual cells. If intact *Gret1* remains, elimination of *Gret1* could continue in these plants. Direct sequencing of 5′-LTR PCR products revealed the G-deletion and the G-insertions at the Gret#4 target site in both alleles in all 19 leaves analyzed (Table 5; examples of the raw sequence data are shown in Fig 3B). In contrast, direct sequencing of 3′-LTR revealed homozygous G deletion in all leaves.

**Table 5:** Mutations in non-eliminated *Gret1* LTR.

Plant Name	leaf No.	5′-LTR		3′-LTR	
		Gret#4 target sequence^¶^	Mutation	Gret#4 target sequence^¶^	Mutation
Wild type		CCTCCT/GGTAACTTCTTAATACC		CCTCCT/GGTAACTTCTTAATACC	No mutation
#67–1	1	CCTCC-GGTAACTTCTTAATACC	T del.	CCTCCT-GTAACTTCTTAATACC	G del.
		CCTCCTGGGTAACTTCTTAATACC	G In.		
	2	CCTCC-GGTAACTTCTTAATACC	T del.		
		CCTCCTGGGTAACTTCTTAATACC	G In.		
	3	CCTCC-GGTAACTTCTTAATACC	T del.		
		CCTCCTGGGTAACTTCTTAATACC	G In.		
	4	CCTCC-GGTAACTTCTTAATACC	T del.		
		CCTCCTGGGTAACTTCTTAATACC	G In.		
	5	CCTCC-GGTAACTTCTTAATACC	T del.		
		CCTCCTGGGTAACTTCTTAATACC	G In.		
	6	CCTCC-GGTAACTTCTTAATACC	T del.		
		CCTCCTGGGTAACTTCTTAATACC	G In.		
	7	CCTCC-GGTAACTTCTTAATACC	T del.		
		CCTCCTGGGTAACTTCTTAATACC	G In.		
#67–2	1	CCTCC-GGTAACTTCTTAATACC	T del.	CCTCCT-GTAACTTCTTAATACC	G del.
		CCTCCTGGGTAACTTCTTAATACC	G In.		
	2	CCTCC-GGTAACTTCTTAATACC	T del.		
		CCTCCTGGGTAACTTCTTAATACC	G In.		
	3	CCTCC-GGTAACTTCTTAATACC	T del.		
		CCTCCTGGGTAACTTCTTAATACC	G In.		
	4	CCTCC-GGTAACTTCTTAATACC	T del.		
		CCTCCTGGGTAACTTCTTAATACC	G In.		
	5	CCTCC-GGTAACTTCTTAATACC	T del.		
		CCTCCTGGGTAACTTCTTAATACC	G In.		
	6	CCTCC-GGTAACTTCTTAATACC	T del.		
		CCTCCTGGGTAACTTCTTAATACC	G In.		
#67–3	1	CCTCC-GGTAACTTCTTAATACC	T del.	CCTCCT-GTAACTTCTTAATACC	G del.
		CCTCCTGGGTAACTTCTTAATACC	G In.		
	2	CCTCC-GGTAACTTCTTAATACC	T del.		
		CCTCCTGGGTAACTTCTTAATACC	G In.		
	3	CCTCC-GGTAACTTCTTAATACC	T del.		
		CCTCCTGGGTAACTTCTTAATACC	G In.		
	4	CCTCC-GGTAACTTCTTAATACC	T del.		
		CCTCCTGGGTAACTTCTTAATACC	G In.		
	5	CCTCC-GGTAACTTCTTAATACC	T del.		
		CCTCCTGGGTAACTTCTTAATACC	G In.		
	6	CCTCC-GGTAACTTCTTAATACC	T del.		
		CCTCCTGGGTAACTTCTTAATACC	G In.		

^¶^ “/” expected cleavage site.

### Detection of mutation in *Gret1*-like sequence in a grafted plant

As *Gret1* is a retrotransposon, multiple copies may exist in the genome. We conducted BLAST searched for finding 20-nt Gret#4 target sequences in the ‘Shine Muscat’ genome using ‘Shine Muscat’ draft genome sequence obtained from Plant GARDEN (https://plantgarden.jp/ja/list/t2599122) as a reference genome [2]. We found 4 Gret#4 target sequences, 2 of them in *Gret1* in the *VvMYBA1a* promoter region. The distance between the other 2 Gret#4 target sequences is the same as the *Gret1* size (9.6kb), so these sequences may be located on the LTRs of another *Gret1*-like sequence. So we analyzed whether the removal of this *Gret1*-like sequence occurred in grafted plant #67–1. This plant showed a band corresponding to 500 bp when Off3-F and Off3-R were used as primers (S1A and S1B Fig, lane 1). Sequencing of this PCR product revealed that the *Gret1*-like sequence, including the 5′-LTR and 3′-LTR, was completely removed from the genome in a part of the plant (S1C Fig). We also sequenced PCR fragments that appeared when we used Off3-F and 5′-LTR-R (S1B Fig, lanes 3, 4) and 5′-LTR-F3IN and Off3-R (lanes 5, 6) to detect any mutations in the 5′-LTR and 3′-LTR in the *Gret1*-like sequence. We found many SNPs in both between *Gret1* on chromosomes 2 and the *Gret1*-like sequences in ‘Shine Muscat’ (S2 and S3 Figs). One of the SNPs corresponds to the PAM sequence, the N portion of NGG, and thus functions as a target for genome editing. Indeed, the #67–1 PCR fragment showed a double peak from the expected cleavage site, indicating that the mutations occurred in some of the cells (S1D Fig). These results mean that the removal of *Gret1*-like sequences and small insertions or deletions at Gret#4 target sites also occurred in a chimeric fashion in #67–1.

## Discussion

Mutations in grape somatic cells accumulated through repeated vegetative propagation create phenotypic diversity in clones. Thus, the economic benefits of clonal selection are large. Among the three types of polymorphism, SNPs, Indels and insertion polymorphism generated by mobile elements of many transposon families displayed the highest mutational event with respect to Pinot noir clonal variation [29]. The position of transposon insertions cannot be controlled, and retrotransposons are rarely eliminated spontaneously. Since transposons can have undesirable effects, there is a need to have techniques for their removal. We used CRISPR/Cas9-mediated LTR cleavage to remove the grape retrotransposon *Gret1*. Recently, Yang et al. [17] used CRISPR/Cas9 editing to remove *Gret1* and restore the function of a *VvMYBA1* allele in *Vitis vinifera* ‘Chardonnay’: CRISPR/Cas9 targeted two sites flanking *Gret1* and they obtained 2 plants with successful *Gret1* removal out of 24 Cas9-positive transgenic plants by biolistic bombardment. However, no or only very faint PCR bands showing *Gret1* removal were observed in Cas9 positive transgenic calluses with *Agrobacterium* method in their study. Interestingly, authors detected several calluses forming solo LTR even though these calluses were non-*MYBA1*-related transgenic samples. Naturally-occurred LTR recombination takes place infrequently but artificial break induction in duplicated LTR sequences drastically increases recombination. In fact, in our experiments, we observed solo LTR structures in 19 of 45 regenerated plants (Table 2). Since it is clear from the example of ‘Ruby Okuyama’ that the presence of the solo LTR does not inhibit fruit skin coloration, cleaving 5′- and 3′-LTR sequences to induce both non-homologous end-joining and homologous recombination seems more efficient for restoring *VvMYBA1* function than cleaving regions flanking LTRs.

For eliminating *Gret1*, selecting a target sequence with high cleavage efficiency in the LTR is important. We compared the DNA cleavage activities of five target sequences, Gret#1 to #5, in an *in vitro* DNA cleavage assay, and found Gret#2, Gret#4, and Gret#5 to be the most efficient. We assessed the stability of a binary vector harboring Cas9, sgRNA, and the selection marker NPTII and detected *Gret1* elimination in Gret#4 and Gret#5, but not Gret#2, transgenic calluses. Because a long culture of transgenic calluses accumulates somaclonal mutations, we obtained transgenic plants targeting Gret#4 and Gret#5 directly from AECs and analyzed *Gret1* elimination by PCR and sequencing. At this stage, SNPs 1 to 4 in all solo LTR fragments (except those with large deletions) were G, A, A, and A respectively (Table 3). These results seem reasonable, because the Gret#4 target site exists between SNPs 1 and 2, and ligation of the two DNA ends creates SNPs G, A, A, and A. On the other hand, in leaves of grafted plants #67–1 to 3, SNPs varied, but there were few variations in solo LTR Gret#4 target sequences (Table 4). These results mean that *Gret1* was eliminated not via non-homologous end joining of the two cleavage ends but via homologous recombination between the 5′-LTR and 3′-LTR regions after regeneration. Mosaic states of SNPs in homologous sequences have been reported in gene targeting experiments, in which homologous recombination occurs between endogenous genomic DNA and extrachromosomal donor DNA [30–33].

We regenerated plants in which *Gret1* was eliminated from the *VvMYBA1a* promoter region and are currently waiting for flowering to check grape berry skin color. However, remnant *Gret1* was also detected in all analyzed leaves, meaning that these genome-edited plants were chimera of both *Gret1* eliminated and non-eliminated cells. To obtain ‘Shine muscat’ fruits with red skin, it will be necessary to obtain non-chimeric plants without *Gret1* in the *VvMYBA1a* promoter region. Plant chimeras may separate by adventitious shoot formation with re-arrangement of the apical cell layers.

In fruit trees, non-chimeric branches can be obtained from secondary or subordinate buds derived from a few apical meristem cells after branch cutting [34]. Another method of obtaining non-chimeric plants is to de-differentiate chimeric tissues and then re-differentiate them. In fact, a microvine was originated through somatic embryogenesis from the L1 layer of ‘Pinot Meunier’ by using anther filaments as explants [35, 36]. Embryogenic calluses or somatic embryos can also be induced from leaves [37]. Using these methods, it should be possible to obtain non-chimeric plants with solo LTRs.

In this study, we reported the establishment of an efficient transposon removal system in grapes. We will continue to grow genome-edited individuals and try to obtain non-chimeric branches for the red skin berry color “Shine Muscat”.

## Supporting information

S1 Fig Detection of mutations in *Gret1*-like sequence. (a) Schematic representation of *Gret1*-like sequence and positions of primers. (b) Detection of solo LTR and non-eliminated *Gret1*-like sequences. Lanes 1,3,5; regenerated plants #67–1; lanes 2,4,6; ‘Shine Muscat’. Primer sets: lanes 1 and 2, Off3-F and Off3-R; 3 and 4, Off3-F and 5’-LTR-R; 5 and 6 are as follows; 5′-LTR-F3IN and Off3-R. (c) Structure of PCR product detected in *b* lane 1. (d) Direct sequences of PCR products in lanes 3 to 6 in *b*. There is a single SNP, indicated in red, in the Gret#4 target sequence in the *Gret1*-like sequence in ‘Shine Muscat’. This sequence is the on-target of Gret#4 sgRNA.
(DOCX)

S2 Fig Alignment of partial 5′-LTR between *Gret1* on chromosome 2 and *Gret1*-like sequence. Upper row, 5′-LTR sequence of *Gret1*; Lower row, 5′-LTR sequence of *Gret1*-like sequence. Blue box, Gret#4 target sequences. Red square, the lack of a matching nucleotide base. #, single nucleotide mismatch. *, Every 10^th^ base is marked with an asterisk (*).
(DOCX)

S3 Fig Alignment of partial 3′-LTR between *Gret1* on chromosome 2 and *Gret1*-like sequence. Upper row, 3′-LTR sequence of *Gret1*; Lower row, 3′-LTR sequence of *Gret1*-like sequence. Blue box, Gret#4 target sequences. Red square, the lack of a mismatching nucleotide base. #, single nucleotide mismatch. *, Every 10^th^ base is marked with an asterisk (*).
(DOCX)

S1 Table Primers used in this study. Primers used for detecting solo LTR and indels in 5′-LTR and 3′-LTR in *Gret1* in *VvMYB1a* promoter region, for *in vitro* cleavage assay, for detecting solo LTR and indels in 5′-LTR and 3′-LTR in *Gret1*-like sequence and for multiplex PCR.
(DOCX)

S1 Raw images (PDF)
