# Supplementary material for: Targeted deletion of grape retrotransposon associated with fruit skin color via CRISPR/Cas9 in Vitis labrascana ‘Shine Muscat’
Source: PLoS One. 2023 Jun 8;18(6):e0286698. doi: 10.1371/journal.pone.0286698 (PMC10249860; doi:10.1371/journal.pone.0286698)
Supplement: S1 Fig — (a) Schematic representation of Gret1-like sequence and positions of primers. (b) Detection of solo LTR and non-eliminated Gret1-like sequences. Lanes 1,3,5; regenerated plants #67–1; lanes 2,4,6; ‘Shine Muscat’. Primer sets: lanes 1 and 2, Off3-F and Off3-R; 3 and 4, Off3-F and 5’-LTR-R; 5 and 6 are as follows; 5′-LTR-F3IN and Off3-R. (c) Structure of PCR product detected in b lane 1. (d) Direct sequences of PCR products in lanes 3 to 6 in b. There is a single SNP, indicated in red, in the Gret#4 target sequence in the Gret1-like sequence in ‘Shine Muscat’. This sequence is the on-target of Gret#4 sgRNA. (DOCX) [file pone.0286698.s001.docx]

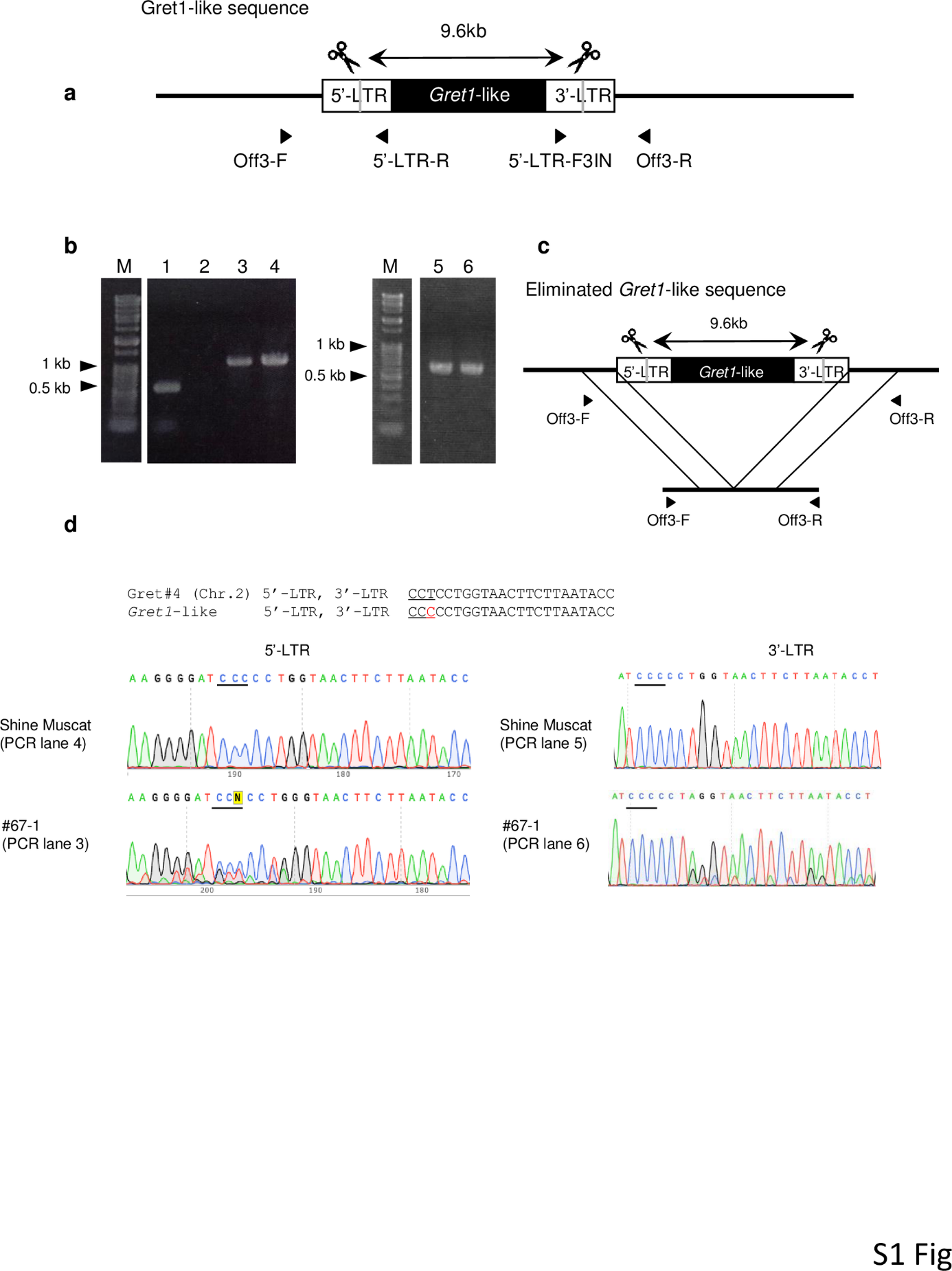


**S1 Fig Detection of mutations in *Gret1*-like sequence.**

(a) Schematic representation of *Gret1*-like sequence and positions of primers.

(b) Detection of solo LTR and non-eliminated *Gret1*-like sequences. Lanes 1,3,5; regenerated plants #67-1; lanes 2,4,6; ‘Shine Muscat’. Primer sets: lanes 1 and 2, Off3-F and Off3-R; 3 and 4, Off3-F and 5’-LTR-R; 5 and 6 are as follows; 5′-LTR-F3IN and Off3-R.

(c) Structure of PCR product detected in *b* lane 1.

(d) Direct sequences of PCR products in lanes 3 to 6 in *b*. There is a single SNP, indicated in red, in the Gret#4 target sequence in the Gret1-like sequence in ‘Shine Muscat’. This sequence is the on-target of Gret#4 sgRNA.
