# Supplementary material for: Targeted deletion of grape retrotransposon associated with fruit skin color via CRISPR/Cas9 in Vitis labrascana ‘Shine Muscat’
Source: PLoS One. 2023 Jun 8;18(6):e0286698. doi: 10.1371/journal.pone.0286698 (PMC10249860; doi:10.1371/journal.pone.0286698)
Supplement: S2 Fig — Upper row, 5′-LTR sequence of Gret1; Lower row, 5′-LTR sequence of Gret1-like sequence. Blue box, Gret#4 target sequences. Red square, the lack of a matching nucleotide base. #, single nucleotide mismatch. *, Every 10th base is marked with an asterisk (*). (DOCX) [file pone.0286698.s002.docx]

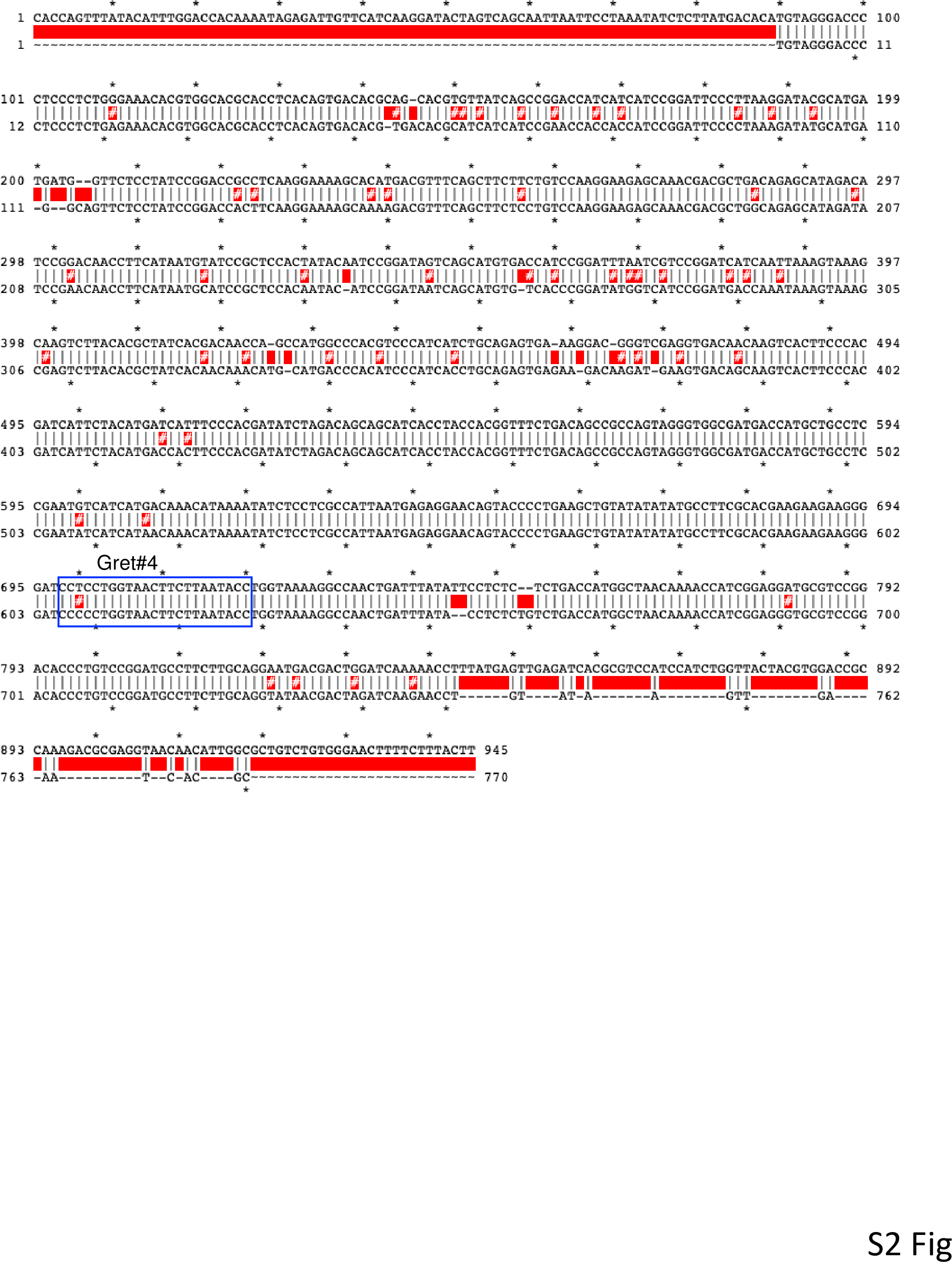
**S2 Fig. Alignment of partial 5′-LTR between *Gret1* on chromosome 2 and *Gret1*-like sequence.**

Upper row, 5′-LTR sequence of *Gret1*; Lower row, 5′-LTR sequence of *Gret1*-like sequence. Blue box, Gret#4 target sequences; Red square, the lack of a matching nucleotide base; #, single nucleotide mismatch; *, Every 10^th^ base is marked with an asterisk (*).
