# Supplementary material for: Targeted deletion of grape retrotransposon associated with fruit skin color via CRISPR/Cas9 in Vitis labrascana ‘Shine Muscat’
Source: PLoS One. 2023 Jun 8;18(6):e0286698. doi: 10.1371/journal.pone.0286698 (PMC10249860; doi:10.1371/journal.pone.0286698)
Supplement: S1 Raw images — (PDF) [file pone.0286698.s005.pdf]

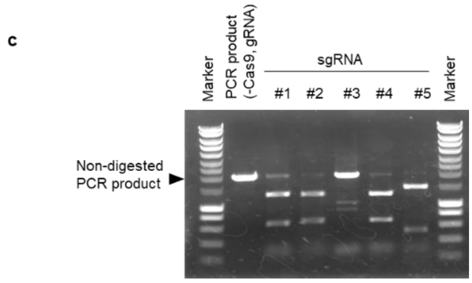

Fig 1. Schematic figure of *VvMYBA1* locus and the peripheral sequences associated with grape skin color.

(**c**) *In vitro* cleavage assay using PCR product, Cas9 protein, and sgRNAs. Black arrowhead: undigested PCR products.

The original image of this figure was shown below.
The images were taken with an ChemiDoc Touch.

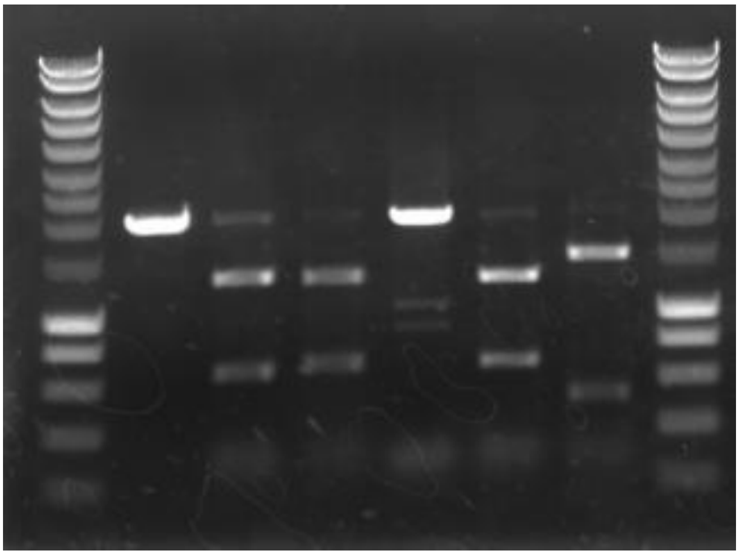

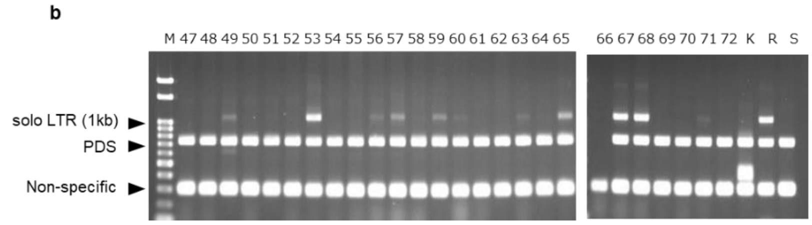

Fig 2. Detection of solo LTR.

(b) Detection of solo LTR in regenerated plants by PCR using primers F1 and R1. Amplification of 1-kb bands means the existence of solo LTR. Primer pairs amplifying *Vitis vinifera* phytoene desaturase (*VvPDS*) were added to amplify internal control. M, DNA size marker; K, 'Kyoho'; R, 'Ruby Okuyama'; S, 'Shine Muscat'.

The original image of this figure was shown below.

The images were taken with an ATTO BIOINSTRUMENT AE-6932XCF.

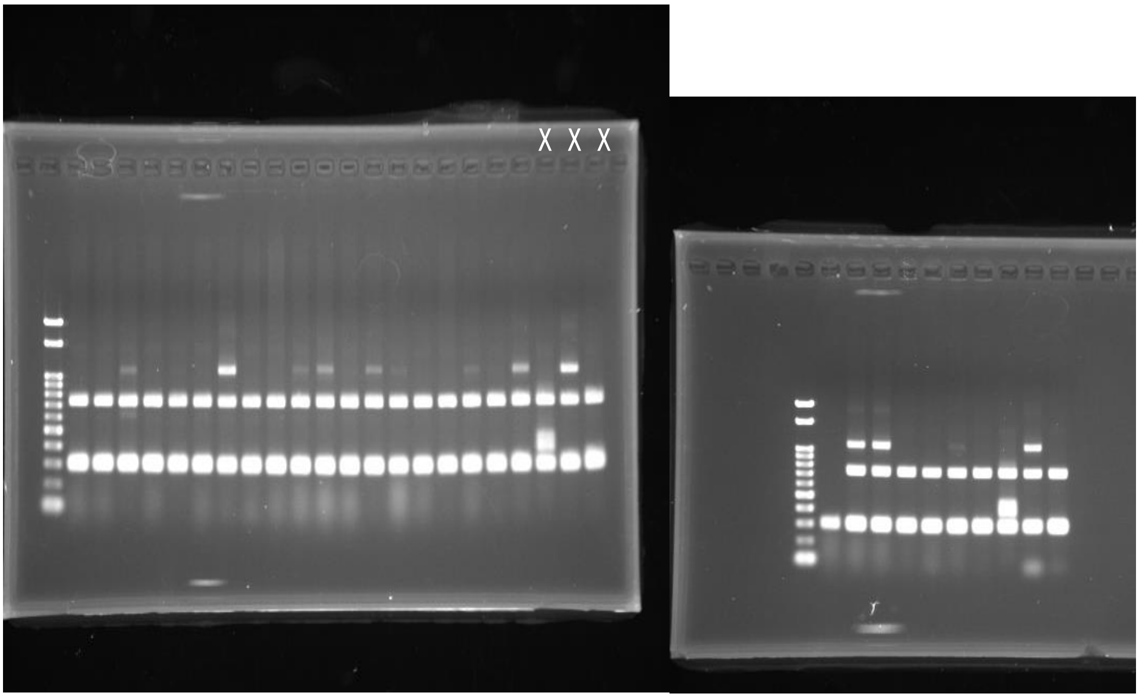

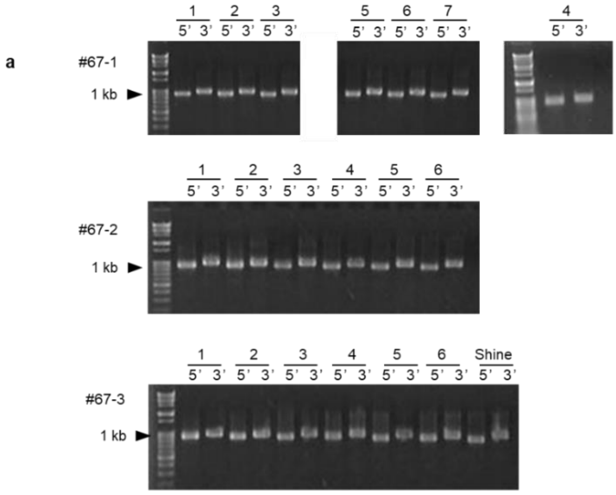

Fig 3 Detection of non-eliminated *Gret1*. (a) PCR amplification of 5′-LTR and 3′-LTR in the leaves of grafted plants #67-1 to 3 using primer sets F1 and 5′LTR-R (5′-LTR), 3′LTR-F and R1 (3′-LTR).

The original image of this figure was shown below. The PCR products from the 4th leaf of plant #67-1 were not detected as shown in XX, so they were electrophoresed separately as shown on the right. The images were taken with an ATTO AE6905H Image Saver HR.

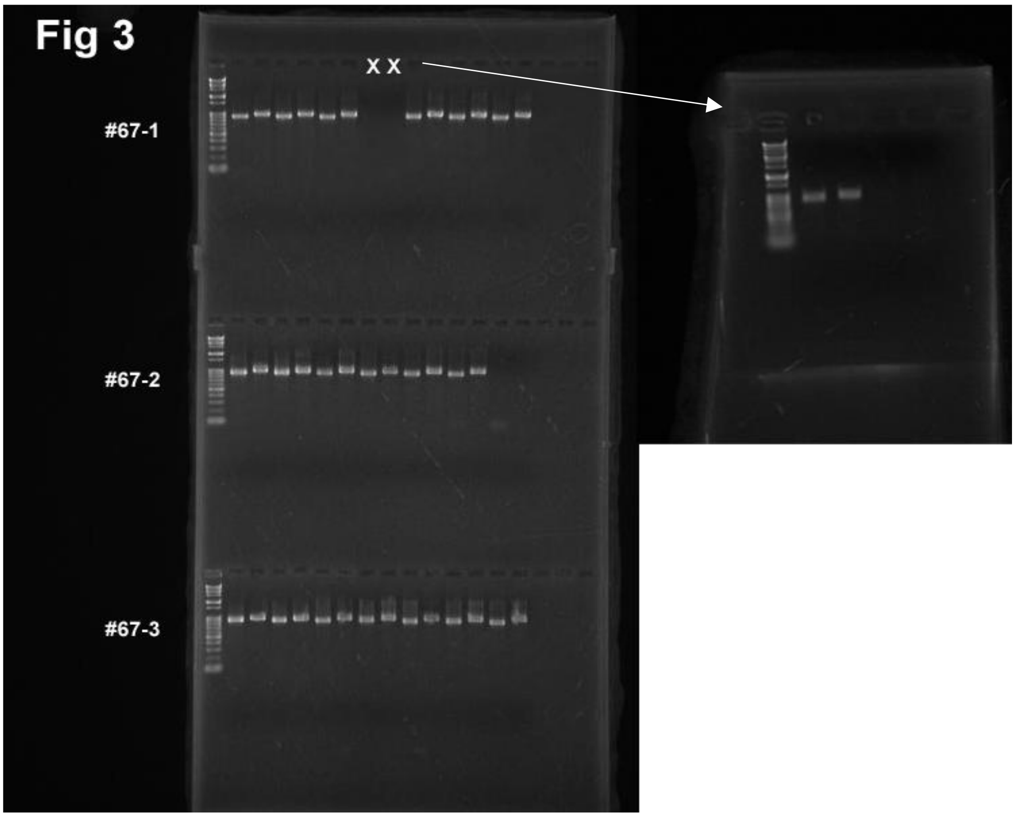

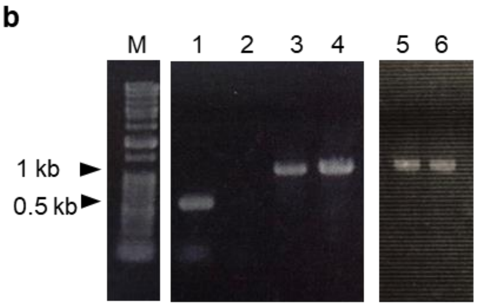

S1 Fig Detection of mutations in *Gret1*-like sequence.

(b) Detection of solo LTR and non-eliminated Gret1-like sequences. Lanes 1,3,5; regenerated plants #67-1; lanes 2,4,6; 'Shine Muscat'. Primer sets: lanes 1 and 2, Off3-F and Off3-R; 3 and 4, Off3-F and 5'-LTR-R; 5 and 6 are as follows; 5′-LTR-F3IN and Off3-R.

As for Figure S1, lanes 1,2,3,4 were taken from the following image. The images were captured using an ATTO AE6905H Image Saver HR.

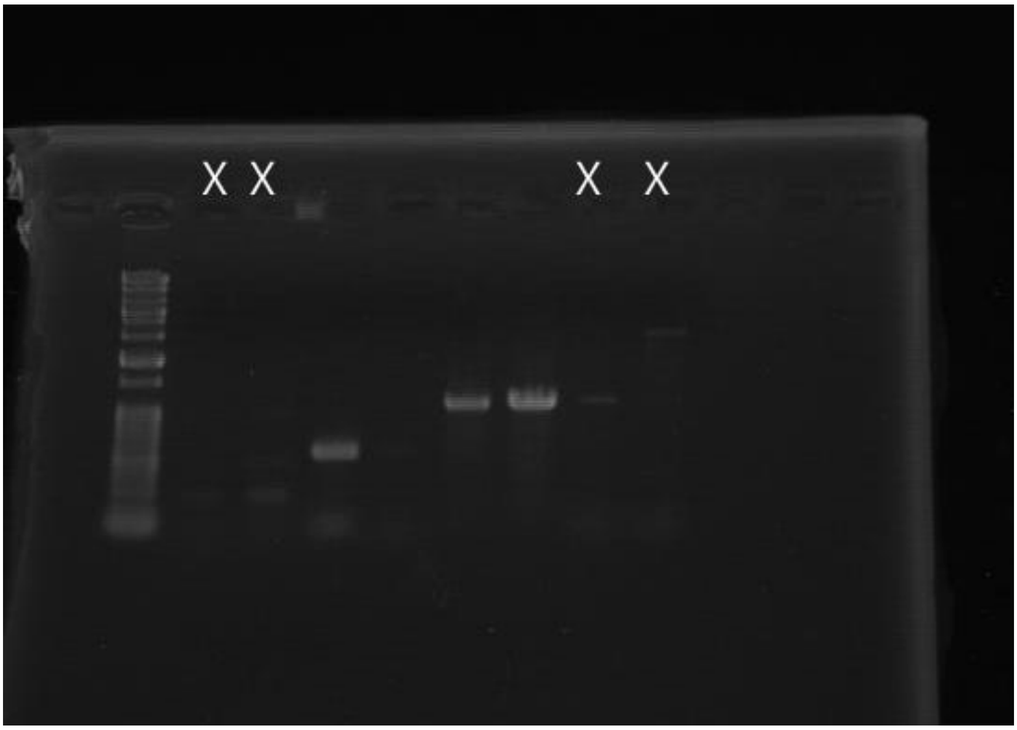

As for Figure S1, lanes 5,6 were taken from the following image. The images were captured using an ATTO AE6905H Image Saver HR, printed, and scanned by Epson GTX-770 scanner.

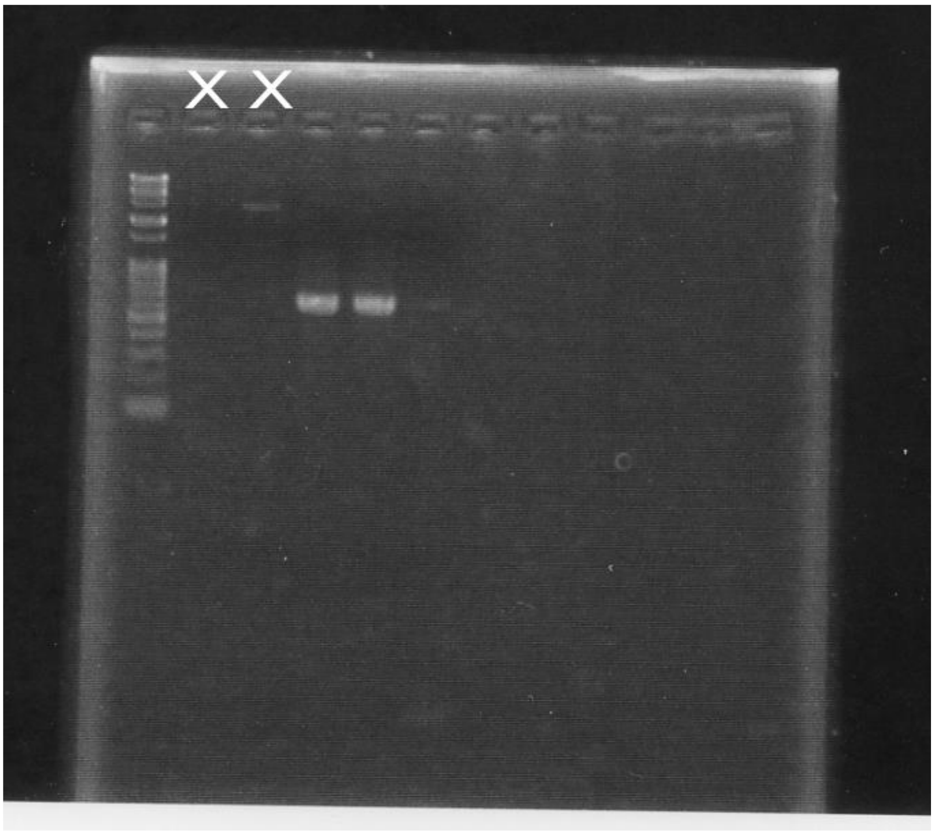
X X
