# Supplementary material for: Targeted deletion of grape retrotransposon associated with fruit skin color via CRISPR/Cas9 in Vitis labrascana ‘Shine Muscat’
Source: PLoS One. 2023 Jun 8;18(6):e0286698. doi: 10.1371/journal.pone.0286698 (PMC10249860; doi:10.1371/journal.pone.0286698)
Supplement: Supplementary file 5 [file pone.0286698.s004.docx]

S1 Table Primers used in this study

Detection of solo LTR and indels in 5′-LTR and 3′-LTR in *Gret1* in *VvMYB1a* promoter region (chromosome 2)

Primer name	Sequence(5′ to 3′)
F1	CACCAGTTTATACATTTGGACCA
R1	GAGACCGCTCCTTCTCTAA
5'-LTR-R	CAATGTTGTTACCTCGCGTCT
3'-LTR-F	ATGTAGGGAACCCCTCCTCT
5'-LTR-F3IN	CTGCCTCCGAATGTCATCAT
3FIN-R	ATGATGACATTCGGAGGCAG

In vitro cleavage assay

Primer name	Sequence (5′ to 3′)
VvMYBA1Pro-F1	AGATATTTCCATAAAACTCGAAAATCAGTGAGGGT
Gret1-R1	TGATCTTCTAGGTTCTTGCGGGCCT-

Detection of solo LTR and indels in 5′-LTR and 3′-LTR in *Gret1*-like sequence

Primer name	Sequence (5′ to 3′)
Off3-F	GTCAAAGTCAAGTAACCACCATG
Off3-R	ATGATTTTGGTCATGGCGGC
5'-LTR-R	CAATGTTGTTACCTCGCGTCT
3'-LTR-F	ATGTAGGGACCCCTCCCTCT
5'-LTR-F3IN	CTGCCTCCGAATGTCATCAT

PDS primers for multiplex PCR

Primer name	Sequence (5′ to 3′)
PDS-MPF1	CTCCTCGTCCCAGTAAACCA
